# A cost function for HIV prevention services: is there a 'u' – shape?

**DOI:** 10.1186/1478-7547-5-13

**Published:** 2007-11-05

**Authors:** Lorna Guinness, Lilani Kumaranayake, Kara Hanson

**Affiliations:** 1 Department of Public Health and Policy, London School of Hygiene & Tropical Medicine, Keppel Street, London, UK, WC1 7HT

## Abstract

**Background:**

Global resource needs estimation is a critical part of addressing the HIV/AIDS epidemic. To generate these estimates knowledge of costs and cost structures is required. The evidence base for costs of HIV prevention programmes is limited. Even less is known about the existence of economies scale and whether, as economic theory suggests, average costs form a 'u'-shaped curve as scale increases. Using an econometric analysis, this paper addresses this question by estimating marginal costs and economies of scale for HIV prevention programmes for vulnerable groups in Southern India with different levels of coverage.

**Methods:**

Two hybrid translog-cost functions were estimated. First, expenditure data from 78 state-funded HIV prevention projects in Andhra Pradesh were used to explore the impact of scale, institutional history and price on costs; second, economic cost data from 16 commercial sex worker projects across Tamil Nadu and Andhra Pradesh were analysed to additionally assess the impact of the value of inputs not reported in expenditure data and location. Coefficient estimates were used to calculate marginal costs and economies of scale.

**Results:**

The econometric model yielded a good fit (R^2 ^= 0.46, p < 0.001 and R^2 ^= 0.79, p < 0.001, for the expenditure and economic cost datasets, respectively). The economies of scale index was greater than 1 for both datasets and fell as coverage increased. Analysis of the expenditure data found economies of scale were not exhausted, with a 0.002% change in total cost for each extra person reached and an 11% difference in total cost between target group categories. Estimation using the economic cost data suggests a point of minimum efficient scale at around 1750–2000 people reached, a 0.03% change in total cost for each extra person reached, and 28% lower costs in Tamil Nadu than Andhra Pradesh.

**Conclusion:**

Econometric analysis of these standardized datasets provides insights into how costs change with coverage, the impact of project location and nature of the project target group. The results demonstrate the importance of understanding the nature of the cost function when designing, budgeting and estimating resource requirements for scaling up coverage of HIV prevention projects.

## Background

Addressing the HIV epidemic is a priority for governments and international agencies throughout the world. A comprehensive prevention package is a core part of this response [1]. Estimates for financing the expansion of HIV prevention services are part of the on-going global resource needs estimation for HIV/AIDS programmes [1,2]. Despite this, the costs and cost structures of HIV prevention programmes are still poorly understood [3-9]. Resource requirement estimates for these programmes rely on scarce evidence and a limited understanding of what and how different factors might influence average costs, especially as programmes are expanded [10-13]. Existing studies show that general health service costs are influenced by scale of activity (the level of output), the production technology applied (the mix of inputs used in service delivery), the scope of activities (e.g. the range of services provided), input prices, levels of efficiency including technical inefficiencies, the context and length of time the service has been provided [14-24]. In the case of scale, economic theory suggests that as output increases average costs will first fall and then rise, resulting in a 'u' – shaped average cost curve. To test such a hypothesis for HIV prevention services estimates of the marginal cost (the change in total cost with each unit increase in scale) using a cost function approach are required [14]. However, standardised data on the cost structures and factors that might influence changes in average costs, needed to carry out this type of analysis, have been lacking [3,4,7-9,20,25].

In India, where 15% of the world's population infected with HIV are living [13], recent studies have started to provide insight into the costs of prevention programmes for commercial sex workers (CSWs), sexually transmitted infections (STI) clinic services, the prevention of mother to child transmission (PMTCT) and voluntary counselling and testing (VCT) services [26-30]. Two of these studies suggest decreasing average costs or economies of scale across the ranges of output examined for CSW and VCT services [26,27]. In contrast, using different data sets, Guinness et al. found, amongst a range of factors, that coverage (number of people reached) explained over 50% of the variation in unit cost of CSW services and, a simple non-parametric regression analysis, suggested a 'u' – shaped average cost curve [28]. Finally, Marseille et al's 2007 multinational study indicates rising cost per 1^st ^visit and cost per mother completing post-test counselling as output increased for STI clinic and PMTCT services in India, respectively [30].

The paper presented here goes beyond the simple regressions used in this previous research to estimate an econometric cost function for HIV prevention services. It uses the CSW cost data presented in Guinness et al [28] and a new set of data from 78 HIV prevention projects for vulnerable groups collected for the present analysis. Marginal costs for different levels of coverage are calculated to measure the degree of scale economies in HIV prevention projects targeted at high risk populations. The impact of other key contextual factors on total and average costs is also assessed.

The paper uses data from HIV prevention projects for vulnerable groups, a priority for the Indian National AIDS Control Programme, in Andhra Pradesh and Tamil Nadu, two Indian states with high levels of prevalence (2.0% and 0.5% of the sampled antenatal clinic population, respectively [31]). The projects are all implemented by local NGOs contracted by the State AIDS Control Societies (SACS) (the Tamil Nadu State AIDS Control Society (TNSACS), Chennai Corporation AIDS Prevention and Control Society (CAPACS) and Andhra Pradesh State AIDS Control Society (APSACS) or other agencies (Christian Council for Rural Development and Research (CCOORR)). The projects comprise a combination of peer education, promotion of safer sex behaviour, referral for treatment of STIs, provision or sales of condoms and creating an environment that facilitates behaviour change, for example working with community leaders and the police.

## Methods

### (i) Data

The sampling frame, sampling methods and data collection instruments are described in Table 1. The sample frame comprised existing HIV prevention projects for vulnerable groups in Tamil Nadu and Andhra Pradesh. Their similar implementation approach (a combination of peer education, condom distribution, referral for STI treatment and creating an enabling environment) is based on the National AIDS Control Programme best practice model [32]. This allows for pooling of the sample across the states and funding agencies.

**Table 1:** Sampling and methods of data collection for the AP financial dataset and case study datasets used in the cost function estimations

	*Financial dataset*	*Case study dataset*^§^
Sample frame	101 NGO HIV prevention projects implementing targeted interventions under contract to APSACS^~^	40 NGO HIV prevention interventions targeted at commercial sex workers and their clients, contracted by CAPACS*, CCOORR**, APSACS^~ ^and TNSACS^#^
Sample size	78 projects from the sampling frame. Exclusion criteria related to non response, missing documentation, mis-reporting in outputs and termination of project	16 purposively selected NGO HIV prevention projects based on geographical location, a range of HIV experience and agency knowledge of quality of services
Data collection instruments	Project reported quarterly expenditure statements submitted to and collected from the management agency alongside a postal survey of NGOs to collate information on coverage and organisational characteristics."	Economic cost and coverage data collected using an ingredients approach based on the UNAIDS costing guidelines [44] during project visits and using routine monitoring records. Costs also include those incurred for technical support, monitoring and contractual management costs at the funding and management agency levels.

^§ ^For more information about this dataset, see [28]; *Chennai Corporation AIDS Prevention and Control Society; **Christian Council for Rural Development and Research; ^~ ^Andhra Pradesh State AIDS Control Society;^#^Tamil Nadu State AIDS Control Society.

Expenditure data, for the financial year 2001/02, from 78 HIV state-funded prevention projects in Andhra Pradesh were analysed (*the financial dataset*) to explore the impact on costs of scale, target group, institutional history and price. This large sample allowed for statistically robust results. However, expenditure data do not provide a complete a picture of costs. They include reported recurrent expenditures which in this case fall in the following categories: rent, personnel, office running expenses, expenses associated with specific activities (behaviour change communication & creating an enabling environment), peer educator incentives, condoms, STI treatment, expenses incurred by staff to participate in training, travel, monitoring and evaluation and "other." They do not include costs incurred at the funding agency level, capital investments or donated inputs. As a result a second smaller dataset (*the case study dataset*) of economic costs, for 2001/02, from 16 commercial sex worker projects across Tamil Nadu and Andhra Pradesh was analysed. This allowed the additional assessment of the impact of costs incurred at the funding agency level and other inputs not valued in the expenditure data, as well as differences across the states. A detailed description of the case study dataset is provided elsewhere [28]. In summary, the additional costs included in the case study dataset are: the annualised capital costs of training, development of educational materials, equipment, furniture and vehicles; monitoring and other managerial costs incurred at the funding agency level; and, donated inputs at the project level, including volunteer time, vehicles and building space used by the projects (see table 2).

**Table 2:** Variables used to represent cost, coverage and prices as well as contextual factors influencing the cost of the projects.

		*Representation in sample (N)*	
		
*Variable*	*Definition*	*Andhra Pradesh financial dataset (n = 78)*	*Case studies (n = 16)*
Total cost	Total annual cost of the project	Annual expenditure of the project for financial year 2001/02 at the NGO level (Source: expenditure statements submitted to the unit managing all NGO projects)	Total annual economic cost including costs incurred at the funding agency level (training, monitoring, other managerial support, supply of educational materials) and value of volunteer time and other donated inputs (Source: primary data collection at NGOs and funding agencies, see also [28]).
Coverage	Number of people within the target community reached by the project in the year of study	Source: postal survey of all projects	Source: NGO monitoring reports
Rent	Annual rent for buildings paid by the NGO for the project	Annual expenditure on rent by the project for the year 2001/02	Annual equivalent market value of building space used by the project.
Target group	High risk group at which project is targeted	Commercial sex workers (n = 17); Men who have sex with men (n = 2); Street children (n = 4); Transgenders (n = 1); Truckers (n = 17); Slum populations (n = 37)	Commercial sex workers (n = 16)
Vulnerable groups	Focus on smaller, relatively static populations allowing for more intensive interactions with the individuals over time	Commercial sex workers (n = 17); Men who have sex with men (n = 2); Street children (n = 4); Transgenders (n = 1)	n/a
Non-vulnerable groups	Focus on larger more mobile populations that are still high risk (i.e. warranting the targeted intervention) so that repeat contacts with individuals are less likely	Truckers (n = 17); Slum populations (n = 37)	n/a
Funding agency (AP financial dataset)	Donor that funded initial recruitment of NGO	Department of International Development (UK) – DFID (n = 30); Andhra Pradesh State AIDS Control Society – APSACS (n = 48)	n/a
Funding agency (case study dataset)	Donor that is currently funding the project	n/a	Tamil Nadu State AIDS Control Society – TNSACS (n = 4); Chennai Corporation AIDS Prevention and Control Society – CAPACS(n = 2);Christian Council for Rural Development and Research – CCOORR (n = 2); APSACS (n = 9)
Agency	Agency that managed initial recruitment of NGO and start up of project, grouped by batch of recruitment to programme	APSACS1 (n = 22); APSACS2 (n = 26); HHP (n = 3); SMA1 (n = 14); SMA2 (n = 13)	n/a
State	Indian state in which project is located	n/a	Andhra Pradesh – AP (n = 9); Tamil Nadu – TN (n = 7)
Age	No. of years the project has been operating	Source: postal survey of all projects	Source: case study analysis

n/a = not applicable; APSACS1 = Andhra Pradesh State AIDS Control Society (APSACS) NGO recruitment batch 1; APSACS2 = APSACS recruitment batch 2; HHP = Department of International Development (UK) (DFID) Healthy Highways Project; SMA1 = DFID State Management Agency (SMA) recruitment batch 1; SMA2 = DFID SMA NGO recruitment batch 2

### (ii) Econometric model specification

The cost function estimation followed methods applied in the hospital cost literature in which total costs are a function of input prices and output [33-36]. To allow for the influence of other explanatory variables beyond output measures, a hybrid functional form was used [33]. This implies that the cost function is linearly homogenous in input prices. It was assumed that the NGOs behave in a cost minimizing way given their constrained budgets.

The equation for the total cost function was therefore:

C=ea0+a1wef(q,x)
 MathType@MTEF@5@5@+=feaafiart1ev1aaatCvAUfKttLearuWrP9MDH5MBPbIqV92AaeXatLxBI9gBaebbnrfifHhDYfgasaacPC6xNi=xI8qiVKYPFjYdHaVhbbf9v8qqaqFr0xc9vqFj0dXdbba91qpepeI8k8fiI+fsY=rqGqVepae9pg0db9vqaiVgFr0xfr=xfr=xc9adbaqaaeGacaGaaiaabeqaaeqabiWaaaGcbaGaem4qamKaeyypa0Jaemyzau2aaWbaaSqabeaacqWGHbqydaWgaaadbaGaeGimaadabeaaliabgUcaRiabdggaHnaaBaaameaacqaIXaqmaeqaaSGaem4DaChaaOGaemyzau2aaWbaaSqabeaacqWGMbGzcqGGOaakcqWGXbqCcqGGSaalcqWG4baEcqGGPaqkaaaaaa@3F48@

Where, C = total cost, a_0 _and a_1 _are constants; q is output of the project, x is a vector of independent variables that shift the cost function and w is a variable representing input prices. The model has a flexible functional form with linear, squared and cubed variables in output. Taking the log of both sides the equation becomes:

ln⁡C=a0+a1w+b1q+b2q2+b3q3+∑i=1jcixi
 MathType@MTEF@5@5@+=feaafiart1ev1aaatCvAUfKttLearuWrP9MDH5MBPbIqV92AaeXatLxBI9gBaebbnrfifHhDYfgasaacPC6xNi=xI8qiVKYPFjYdHaVhbbf9v8qqaqFr0xc9vqFj0dXdbba91qpepeI8k8fiI+fsY=rqGqVepae9pg0db9vqaiVgFr0xfr=xfr=xc9adbaqaaeGacaGaaiaabeqaaeqabiWaaaGcbaGagiiBaWMaeiOBa4Maem4qamKaeyypa0Jaemyyae2aaSbaaSqaaiabicdaWaqabaGccqGHRaWkcqWGHbqydaWgaaWcbaGaeGymaedabeaakiabdEha3jabgUcaRiabdkgaInaaBaaaleaacqaIXaqmaeqaaOGaemyCaeNaey4kaSIaemOyai2aaSbaaSqaaiabikdaYaqabaGccqWGXbqCdaahaaWcbeqaaiabikdaYaaakiabgUcaRiabdkgaInaaBaaaleaacqaIZaWmaeqaaOGaemyCae3aaWbaaSqabeaacqaIZaWmaaGccqGHRaWkdaaeWbqaaiabdogaJnaaBaaaleaacqWGPbqAaeqaaOGaemiEaG3aaSbaaSqaaiabdMgaPbqabaaabaGaemyAaKMaeyypa0JaeGymaedabaGaemOAaOganiabggHiLdaaaa@5676@

Using coefficient estimates, the marginal cost of output is:

*MC *= *C *(*b*_1 _+ 2*b*_2_*q *+ 3*b*_3_*q*^2^)

The measurement of marginal cost allows for the calculation of an index of economies of scale (EOS). Following Weaver and Deolalikar (2004) and Barnum and Kutzin (1993) the derived equations for economies of scale are therefore [14,36]:

EOS=1−σc,k∑i=1jσC,qi
 MathType@MTEF@5@5@+=feaafiart1ev1aaatCvAUfKttLearuWrP9MDH5MBPbIqV92AaeXatLxBI9gBaebbnrfifHhDYfgasaacPC6xNi=xI8qiVKYPFjYdHaVhbbf9v8qqaqFr0xc9vqFj0dXdbba91qpepeI8k8fiI+fsY=rqGqVepae9pg0db9vqaiVgFr0xfr=xfr=xc9adbaqaaeGacaGaaiaabeqaaeqabiWaaaGcbaGaemyrauKaem4ta8Kaem4uamLaeyypa0tcfa4aaSaaaeaacqaIXaqmcqGHsisliiGacqWFdpWCdaWgaaqaaiabdogaJjabcYcaSiabdUgaRbqabaaabaWaaabCaeaacqWFdpWCdaWgaaqaaiabdoeadjabcYcaSiabdghaXnaaBaaabaGaemyAaKgabeaaaeqaaaqaaiabdMgaPjabg2da9iabigdaXaqaaiabdQgaQbGaeyyeIuoaaaaaaa@4611@

Where EOS is the economies of scale index, *σ*_*a*_,_*b *_is the elasticity of a with respect to b and k is the capital stock. For this set of cross-sectional data with a single output and in which variations in capital stock have been controlled for (see below), this simplifies to:

EOS=1Q(b1+2b2q+3b3q2)
 MathType@MTEF@5@5@+=feaafiart1ev1aaatCvAUfKttLearuWrP9MDH5MBPbIqV92AaeXatLxBI9gBaebbnrfifHhDYfgasaacPC6xNi=xI8qiVKYPFjYdHaVhbbf9v8qqaqFr0xc9vqFj0dXdbba91qpepeI8k8fiI+fsY=rqGqVepae9pg0db9vqaiVgFr0xfr=xfr=xc9adbaqaaeGacaGaaiaabeqaaeqabiWaaaGcbaGaemyrauKaem4ta8Kaem4uamLaeyypa0tcfa4aaSaaaeaacqaIXaqmaeaacqWGrbqucqGGOaakcqWGIbGydaWgaaqaaiabigdaXaqabaGaey4kaSIaeGOmaiJaemOyai2aaSbaaeaacqaIYaGmaeqaaiabdghaXjabgUcaRiabiodaZiabdkgaInaaBaaabaGaeG4mamdabeaacqWGXbqCdaahaaqabeaacqaIYaGmaaGaeiykaKcaaaaa@43AF@

Economies of scale are the gains in efficiency associated with the level of output. If EOS is greater than one then the level of output is less than the most efficient level. If it is less than one the level of output is greater than the most efficient level of output. When EOS is equal to one there are constant returns to scale.

### (iii) Variables

Table 2 describes the variables used in the model. The single output measure (q) is coverage (the number of members of the target group reached i.e. individuals that have received HIV prevention services from the project). Other available measures of output (number of contacts, treated STIs) are directly related to the level of coverage and each other. If entered into the model they could cause problems of multicollinearity. A single price variable (w) was entered into the model (*rent*), representing regional variations in prices of locally purchased goods. Prices of personnel, drugs and condoms were not needed as they are set centrally and are constant across the projects [35]. As input substitution across other inputs (training, building and office expenses, monitoring and evaluation etc) is limited, it was valid to exclude all prices except for one, the cost of rent (*rent*) representing regional variations in prices of locally purchased goods[35,37,38]. Although, this variable is used in the summation of total costs, its correlation coefficient with total cost was less than 0.6 (Spearman's R = 0.5166, p < 0.001), indicating the relationship was insufficient to cause major bias.

Contextual factors likely to influence costs included were target group (*target group*), contractual history (*funding agency/agency*), location of the project (*state*) and project age (*age*) (see table 2). *Target group *influences costs as some populations are more difficult to reach than others. *Funding agency/agency *captures variations in start-up input, ongoing training and technical support and so controls for differences in capital stock (see table 2). *State *was included to examine the influence of the different settings on cost. Finally, *age *can lower average costs through learning or increase average costs as more experienced workers demand higher salaries.

### (iv) Estimation

The models were estimated using Stata version 8 and ordinary least squares regression. Following equation (2), the model was first estimated with the linear, squared and cubic coverage terms:

ln *C *= *a*_0 _+ *a*_1_*w *+ *b*_1_*q *+ *b*_2_*q*^2 ^+ *b*_3_*q*^3 ^+ *c*_1_*x*_1 _+ *c*_2_*x*_2 _+ *c*_3_*x*_3 _+ *c*_4_*x*_4_,

The regressions with higher order terms in coverage were potentially collinear causing instability in the estimates. The mean variance inflation factor (VIF) was used to identify multicollinearity. If the mean VIF for a model is greater than 1, multicollinearity is said to be a problem [39]. In the cubic models, the mean VIF's were 503.2 and 79.17 for the case study and financial datasets, respectively. The joint significance of squared and cubic terms in coverage was tested and found to be insignificant for both datasets and omitted from the model. A non-significant result from Ramsey's RESET test for the financial data set indicated that the new specification was correct (H0: the model has no omitted variables: F = 0.35, p = 0.9056). The results of the Ramsey RESET test for the case studies were more ambiguous (H0: the model has no omitted variables: F = 3.95, p = 0.0594). The presence of higher order terms could be rejected at the 95% confidence level. To examine this further, additional analyses were undertaken retaining the squared and cubic terms left in the model. Multicollinearity appeared to affect the estimation by generating the wrong sign on coverage squared, leading to negative values of marginal cost. This supported the choice to exclude the squared and cubic terms on coverage from the estimation.

The linear version was run with different combinations of the dummy variables listed in Table 2 and using both direct values and subsequently natural logs of the independent variables. The F statistic on all models run was significant at the 95% confidence level. The best fit model was therefore selected based on the value of the adjusted R^2 ^and whether coefficients on the independent variables were significant (p < 0.10).

### (v) Identifying the best fit model

For the financial dataset, *target group *was the only factor beyond price and coverage found to influence the total cost function. Assuming that *agency/funding agency *is a good indicator of the variation in start up costs, the lack of significance of this variable was interpreted as meaning that start up costs had little influence on variable costs. As other capital investment, was minimal, no further control for capital stock was considered to be required. The final model specification for the financial dataset was therefore:

ln *C *= *a*_0 _+ *a*_1 _In *w *+ *b*_1_*q *+ *c*_1_*x*_1_,

Where x_1 _is a dummy variable representing vulnerable group interventions (non-vulnerable group being the excluded category).

For the case studies, *state *was the only other factor influencing the cost function significantly. Good fits were obtained with the direct values of price as well as their natural log. To facilitate comparability with the analysis of the financial dataset, the natural log of the price variable was used. The specification for the case study dataset was therefore:

ln *C *= *a*_0 _+ *a*_1 _In *w *+ *b*_1_*q *+ *c*_1_*x*_1_

Where x_1 _is a dummy variable representing the state of Tamil Nadu (Andhra Pradesh being the excluded category).

The Cook-Weisberg (Breusch-Pagan) test was used to test for heteroscedasticity. This was found to be absent in the final models. Marginal cost and economies of scale were calculated using equations (3) and (5), respectively, with the predicted value of cost. To transform the geometric mean of the logged dependent variable to the arithmetic mean of the original variable, the average of the exponential of the residuals was used as a smearing factor so that [40-42]:

E(C)=ea0+a1wef(x,q)emean(resid)
 MathType@MTEF@5@5@+=feaafiart1ev1aaatCvAUfKttLearuWrP9MDH5MBPbIqV92AaeXatLxBI9gBaebbnrfifHhDYfgasaacPC6xNi=xI8qiVKYPFjYdHaVhbbf9v8qqaqFr0xc9vqFj0dXdbba91qpepeI8k8fiI+fsY=rqGqVepae9pg0db9vqaiVgFr0xfr=xfr=xc9adbaqaaeGacaGaaiaabeqaaeqabiWaaaGcbaGaemyrauKaeiikaGIaem4qamKaeiykaKIaeyypa0Jaemyzau2aaWbaaSqabeaacqWGHbqydaWgaaadbaGaeGimaadabeaaliabgUcaRiabdggaHnaaBaaameaacqaIXaqmaeqaaSGaem4DaChaaOGaemyzau2aaWbaaSqabeaacqWGMbGzcqGGOaakcqWG4baEcqGGSaalcqWGXbqCcqGGPaqkaaGccqWGLbqzdaahaaWcbeqaaiabd2gaTjabdwgaLjabdggaHjabd6gaUjabcIcaOiabdkhaYjabdwgaLjabdohaZjabdMgaPjabdsgaKjabcMcaPaaaaaa@518A@

## Results

### (i) Descriptive statistics

Tables 3 and 4 provide descriptive statistics for the datasets. Forty CSW projects had been identified across the two states. For the case study data, 16 were selected purposively based on location, experience and quality of services (see table 1). On average the NGOs selected were smaller than the total population of projects identified in terms of total organisation annual expenditures (INR 2.2 million and INR 4.5 million, respectively) and total staff numbers (27 and 46, respectively). They had a similar level of experience in the field of HIV, with an average of 5 years working on HIV prevention. It is possible that there is a bias towards better quality projects as NGOs' funders were unlikely to invite the research team to poorer quality sites. This could have led to higher total costs and coverage.

**Table 3:** Sample means of the annual economic cost, coverage, project age and annual rent paid by state and funding agency, from the economic costing of the case studies, N= 16 (range)

		State			Funding agency				
		
		*Andhra Pradesh*	*Tamil Nadu*	t test for difference, p value	CAPACS*	CCOORR**	*APSACS*^~^	*TNSACS*^#^	ANOVA for difference, p value
	Total								
Annual economic cost, INR	956,641 (474,299–2,220,988)	1,222,151 (839,664–2,220,988)	615,270 (474.300–775,062)	0.0016^^^	726,320 (68,931)	503,776 (41,685)	1,222,151 (439,036)	610,167 (84,761)	0.059^
No. of people reached	1,165 (205–2008)	1,523 (935–2008)	704 (250–1749)	0.0015^^^	700 (212)	281 (43)	1,523 (375)	989 (750)	0.173
Cost per person reached, INR	1,011.1 (414.5–2,133)	806.3 (563.2–1,106.1)	1,274.4 ((414.5–2,133)	0.9675	1071.9 (911.8–1232.0)	1829.0 (152501–2133.0)	806.3 (563.2–1106.1)	1039.5 (414.5–2082.5)	0.026
Project age, yrs	7.1 (3.3–13.0)	7.00 (3–13)	7.29 (4–12)	0.5628	9.00 (4.2)	5.50 (0.7)	7.00 (3.8)	7.00 (2.9)	0.596
Rent	48,660 (15,135–80,700)	57,525 (30,697–77,840)	37,262 (15,135–80,700)	0.0261^^	58,350 (31,608)	17,468 (3,298)	57,525 (16,857)	37,650 (5,529)	0.202
Sample size	16	9	7		2	2	8	4	

*Chennai Corporation AIDS Prevention and Control Society; **Christian Council for Rural Development and Research; ^~ ^Andhra Pradesh State AIDS Control Society; ^#^Tamil Nadu State AIDS Control Society; ^^^significant at the 99% confidence level; ^^significant at the 95% confidence level; ^significant at the 90% confidence level.

**Table 4:** Sample means of the annual expenditure, coverage, project age and annual rent paid by target group and agency, from the Andhra Pradesh financial dataset (N = 78) (range)

		Target group*			"Agency": Management agency at recruitment of NGO & batch of recruitment **					
		
	Total	*Non-vulnerable*	*Vulnerable*	t test, p value	*APSACS1*	*APSACS2*	*HHP*	*SMA1*	*SMA2*	ANOVA p value
Total expenditure INR 000s	689.2 (40.85–1,581)	663.8 (40.85–1,581)	746.3 (528.1–1,246.9)	0.9375	677.1 (40.9–1,457.3)	639.2 (418.3–1,581)	822.2 (474.4–1,091)	767.3 (467.5–1,247)	695.0 (528.1–1,046)	0.185
No. of people reached	5,647 (675–24,111)	6,809 (993–24,111)	3,034 (675–14,871)	0.0004^^^	6,611 (993–13,955)	6,064 (1,027–17,614)	18,624 (14,390–24,111)	3,479 (1,555–14,871)	2,525 (675–7,099)	0.255
Expenditure per person reached, INR	224.5 (11.6–939.1	143.0 (11.6–592.6)	408.0 (74.3–939.1)	1.000	139.5 (11.6–529.0)	147.7 (35.4–592.6)	46.8 (27.3–75.8)	314.8 (74.3–748.4)	466.0 (107.0–939.1)	0.000^^^
Project age, yrs***	2.72 (2.1–5.6)	2.60 (2.1–5.6)	3 (2.1–3.92)	0.9724	2.5 (0)	2.25 (0)	5.59 (0)	3.92 (0)	2.08 (0)	n/a
Rent, INR	48,480 (0–90,338)	44,937 (0–71,540)	56,452 (31,399–90,338)	0.997	44,542 (0–61,891)	45,057 (19,905–65,388)	48,699 (29,680–71,541)	56,164 (31,281–72,677)	53,664 (43,059–90,338)	0.168
Sample size	78	54	24		22	26	3	14	13	

*"Vulnerable group" projects include those for commercial sex workers (CSW), street children (SC), transgenders (TG) and men who have sex with men (MSM); "Non vulnerable group" projects are those for truckers, mobile populations and slum dwellers. ** Prior to 2001, the targeted interventions were funded and managed by 3 different projects – Andhra Pradesh State AIDS Control Society (APSACS); Department for International Development UK (DFID) supported Healthy Highways Project (HHP); and the DFID supported State Management Agency (SMA). The dummies represent these 3 different projects and that both APSACS and SMA recruited NGOs in 2 separate batches.***Projects started at the same time within each "agency" group. ^^^significant at the 99% confidence level; ^^significant at the 95% confidence level; ^significant at the 90% confidence level.

Within the case study data, there is a significant difference in mean annual economic cost across *state *(p < 0.01) and *funding agency *(p < 0.10). *Coverage *is significantly different across *state *but not across *funding agency*. Average cost is found to vary significantly across *funding agency *but not *state*. There is also a significant difference in *rent *between the two states.

The sampling frame for the financial dataset comprised all 101 projects supported by APSACS at the time of the study. Of these it was found that 5 were no longer in operation, documentation was missing for a further 3 and a further 11 failed to respond to our postal survey. In addition, in 4 cases, reported coverage variables were in unrealistic ranges relative to the town or district population size, and so were excluded from the study. Due to the lack of documentation it is not possible to assess whether there is a systematic difference between these 23 projects and the 78 finally included in the sample.

In the financial dataset no significant difference across target groups or *agency *was found for expenditure, age, or rent. Only *coverage *varied significantly across the target groups and only average expenditure varied across the *agency variable*. It appears that there is also a tendency for total expenditure and rent to vary across the *agency *variable (p = 0.185 and 0.168, respectively).

The case study data yielded a mean annual cost of INR 956,641 and coverage of 1,165 as compared with a total annual expenditure of INR 689,209 and coverage of 5,647 for the financial data (1 USD = 43.53INR [43]). The mean cost/expenditure per person were INR1,011 and INR 225 for the case study and financial datasets respectively. The mean annual cost and cost per person reached in the case study data are 1.4 times and 4.5 times the mean annual expenditure and expenditure per person derived from the financial data, respectively.

### (iii) Cost function estimates

#### Goodness of fit

Results from the best-fit regressions for each dataset are presented in table 5. For the financial dataset, the adjusted R^2 ^is 0.46 and the F statistic is significant at the 99% confidence level (F = 22.48, p < 0.001). For the case study dataset the adjusted R^2 ^is 0.79, with a strongly significant F test (F = 19.71, p < 0.001).

**Table 5:** Cost function estimates

*Variable*	*AP financial data*		*Case studies*	
	*Coefficient*	*Standard error*	*Coefficient*	*Standard error*

Constant	8.053843*	0.909046	11.55254*	0.718119
Ln(price)	0.4827907*	0.0849852	0.1729654**	0.0627868
Coverage	2.01 × 10^-5 ^*	4.52 × 10^-6^	0.000347**	0.000127
Vulnerable group	0.1143**	0.04873		
Tamil Nadu			-0.2801**	0.12508
N	76^§^		16	
F	22.48*		19.71*	
Adjusted R^2^	0.4622		0.7891	
Test for heteroscedasticity (*χ*^2^(1))	5.81**		3.29***	
Additional tests				

*significant at 99%; **significant at 95%; ***significant at 90%; ^§ ^Two cases are dropped as rent = 0

#### Relationship between coverage and total cost

For each model, the coefficient on coverage is statistically significant. The relative impact of scale on cost varies across the datasets. There is a 0.03% change in total cost for each extra person reached in the case study dataset, compared with a 0.002% change in total cost for each extra person reached in the financial dataset.

#### Marginal costs

**Figure 1:**
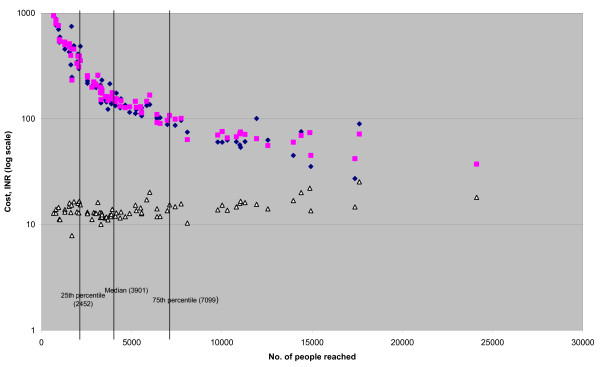
**Average, predicted average and marginal cost, INR, of targeted HIV prevention projects – financial dataset**. Blue diamond: Actual average cost. Pink square: Predicted average cost. White triangle: Marginal cost.

**Figure 2:**
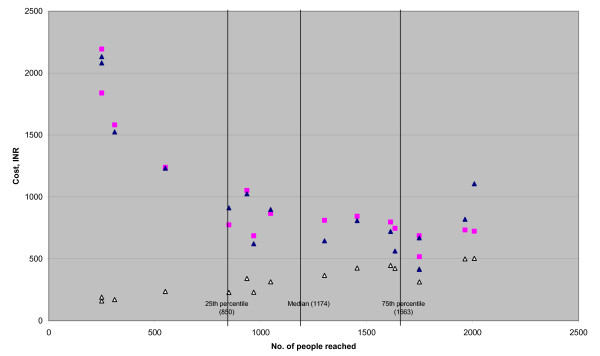
**Average, predicted average and marginal cost, INR, of targeted HIV prevention projects – case study dataset**. Blue diamond: Actual average cost. Pink square: Predicted average cost. White triangle: Marginal cost.

The marginal cost at the median level of coverage in the case study dataset is over 25 times the equivalent value for the financial dataset (INR335 and INR 13, respectively). The higher value in the case study data is likely due to the different shapes of the cost functions as well as the nature of the data (economic cost in the former and financial cost in the latter). Marginal cost also varies across coverage levels within each dataset. For the financial dataset (median coverage = 3,901) the marginal costs are INR 14.26, INR 13 and INR 15.4 at the 25^th ^percentile, median and 75^th ^percentile of coverage, respectively. Figure 1 shows that marginal costs increase over the range of coverage for the financial dataset. For the case studies (median coverage = 1,174 people reached), marginal cost also rises as coverage increases (see figure 2): marginal cost at the 75^th ^percentile of coverage is 1.9 times that at the 25^th ^percentile of coverage (INR 450 and INR 227, respectively).

#### Average cost

Figures 1 and 2 also describe the shape of the predicted and actual average cost curves. For the financial dataset, average costs decline at a decreasing rate over the coverage range. There is an approximately four-fold drop in costs between coverage levels of 1000 and 5000 people reached. Average costs are then halved from INR 129 to INR 65 as coverage increases from 5000 to 15000. Average costs do not reach a minimum, nor does the marginal cost curve cross the average cost curve. On the other hand, average costs in the case study sample suggest there is some minimum efficient scale of operation for commercial sex worker projects. The average cost is 2.4 times higher at 500 people reached than at its lowest point at 1750 people reached, falling from INR 1231 to INR 516. Average cost then rises again to 727 INR at 2000 people reached.

#### Economies of scale

EOS is greater than 1 for both the datasets across the interquartile range of coverage. This indicates that economies of scale are not exhausted over this output range. The EOS falls from 21.29 (CI = 14.02–36.75) for the 25^th ^percentile value of coverage (2451), to 7.01 (CI = 4.82–12.69) at the 75^th ^percentile (7099). The fall in EOS as coverage increases, as well as the shape of the average cost curve, also suggests that as coverage increases the projects are moving towards greater scale efficiency (see figure 1). For the case study data the economies of scale appear to be exhausted within the range of coverage analysed (at the maximum value of coverage (2008), EOS = 1.4 (CI = 0.8–5.6)).

#### Impact of non-scale factors on cost

The estimation shows that costs also vary with location and target group. For the case studies, the coefficient on *state *is negative and significant (p = 0.045), i.e. costs are 28% lower in Tamil Nadu than Andhra Pradesh. For the financial dataset, including *target group *improves model fit (p = 0.0220), indicating that the "vulnerable" group interventions are 11% higher in total cost than the "non-vulnerable" group interventions.

Price also has a positive and significant relationship with total cost (p = 0.017 for the case study dataset, p < 0.001 for the financial dataset). Costs are therefore price inelastic so that a 1% increase in price leads to a rise in costs of 0.48% and 0.17% increase in costs in the financial and case study datasets, respectively.

The model including *agency *was rejected as the adjusted R^2 ^was lower than in the model including *state*. However the high adjusted R^2 ^and significance of the model (adjusted R^2 ^= 0.7887, p < 0.001) led to the perception that *agency *could impact on costs. The rejected model was therefore run using the case study data i.e. including *agency *and excluding *state*. The new model found total costs of the TNSACS and CAPACS projects were 54% (p = 0.021) and 47% (p = 0.043) less than the APSACS projects, respectively.

## Discussion

This paper has presented the econometric estimation of a cost function for HIV prevention services using two sets of data. Results generated from the datasets are similar: there are differences in costs across the targeted interventions associated with scale and local prices. For the case study data, the analysis found that there were scale efficiencies to be exploited. These appear to be exhausted at a coverage level of between 1750–2000 CSWs reached. Although the up turn in the average cost curve is driven by only two data points, the EOS index shows projects with higher levels of coverage are operating with close to constant returns to scale. The model based on the financial dataset found that economies of scale are not exhausted even at higher levels of coverage. In addition, differences between target groups are an important influence on cost. Total cost of vulnerable group projects are on average 11% higher than for the non-vulnerable group. This change in the intercept in the relationship between cost and coverage implies higher fixed costs in the vulnerable group projects. This is likely to reflect greater difficulty in reaching the more marginalized groups represented in vulnerable group projects (e.g. CSWs, men who have sex with men) and requiring greater investment in initiating the project, in particular in establishing a relationship with the community. When agency is included in the model, the case study data also confirms a difference in the production costs between funding agencies.

This analysis represents the first of its kind for HIV prevention programmes. However, it does have limitations. In both cases, the datasets are cross-sectional. This precludes examination of time effects on cost e.g. lagged cost or lagged coverage and leads to a possible bias in results. The quality of the case study data has been discussed elsewhere[28]. The small size of this data set could affect the level of significance of the different models tested in the selection of the best fit. Despite the limited data, the F statistic on all models was significant at the 95% confidence level. The financial dataset is subject to misreporting. However as the NGOs are unable to exceed the agreed budget this is most likely to affect the proportion of spending on individual line items rather than the total reported expenditure used here. In the present analysis, coverage is considered to be exogenous. Targets are frequently not met and are based on estimates of target population size. In addition, although budgetary guidelines, issued by the National AIDS Control Programme, have the potential to restrict flexibility [32], it is believed that there is sufficient variation in costs to warrant the econometric approach. Budget setting involves NGO consultation, consideration of the previous years' activities and the budgets granted deviate from the guidelines. A concern remains that pre-determined costs and outputs lead to bias in the regression coefficients [35].

The results from the financial and case study datasets are striking for both their similarities and differences. The best-fit functional form is almost identical across the two datasets. Both display some potential for economies of scale. There is a major difference in the coefficients on coverage in the regressions, i.e. the proportionate change in cost associated with an additional person reached. This results in the steeper marginal cost curve generated from the case study data. Along with the 'u' – shape of the average cost curve, seen in the case study data analysis, this could arise from a number of factors. First, the range of coverage in the financial dataset (675–24,111 people reached) is far greater than in the case study dataset (250–2008 CSWs reached). Second, definitions of total cost vary. The economic costing (case study dataset) incorporates the value of volunteer time, the value of all inputs irrespective of the funding source and the inputs of training, monitoring and supervision and management made by the funding or management agency. Together these contain the value of a number of fixed inputs. Theoretically, it is fixed costs that are responsible for increases in average costs as scale increases. As a result, the inclusion of these fixed costs in the case study dataset is likely to have important implications for the differences in marginal cost and economies of scale as coverage increases across the datasets. Third, it may be harder to reach CSWs beyond the limit of the population within a specific geographical location, giving rise to an increase in fixed costs. Finally, the appropriate functional form for the case studies may not in fact coincide with that for the financial dataset. The more ambiguous results of the Ramsey RESET test for the case study model indicate that either higher order terms or interactions of the dummies with output may have been omitted. However, persistent problems with multicollinearity did not permit stable estimation with higher order terms and the small sample precluded the inclusion of interaction terms.

Kumaranayake and Watts (2000), using cost data from a range of HIV/AIDS prevention and care programmes, found that projects are likely to encounter diseconomies of scale arising from infrastructural barriers as coverage increases [5]. The analysis of the case study data reinforces these more general findings and suggests a point of minimum efficient scale. On the other hand, results from the financial dataset indicate continuing economies of scale, at least over the range examined here. These support the findings of Dandona et al in examining the economics costs of HIV prevention for commercial sex workers also in India (coverage range = 803–6379) [26]. The differing results could be associated with the wider range of coverage. They also suggest that the case study analysis may reflect the impact of bottlenecks that can be addressed in the long run. If this is the case, the coverage level at which fundamental changes in the fixed costs are required in order to improve efficiency as activities are scaled up is therefore around 1750–2000. The paper adds to a growing literature which finds contrasting results on costs and scale in the area of HIV prevention [5,26-28,30]. These differences and the influence of the contextual factors, identified here, underline the importance of full economic costing and the understanding of a project's context when planning and estimating resource requirements.

## Conclusion

This paper presents the estimation of a cost function for HIV prevention services using two datasets, using a flexible functional form. The combination of the two standardised datasets and econometric techniques has provided greater insights into how costs change with coverage and the key factors that influence total costs. The findings indicate there are economies of scale as coverage increases. The case study dataset suggests that, in terms of scale efficiency, it would be optimal for a project to operate at a coverage in the region of 1750–2000 sex workers. The financial dataset suggest that large scale projects are more efficient than small scale projects with a 5 fold increase in coverage level (1000 to 5000 people reached) leading to four fold drop in average cost. The results also show that local price variations, the project target group and location are important influences on average cost. The analysis demonstrates the importance of understanding the nature of the cost function in designing project contracts, selecting efficient levels of coverage for these projects, constructing their respective budgets and for estimating resource requirements for scaling up coverage of HIV prevention projects.

## Abbreviations

APSACS Andhra Pradesh State AIDS Control Society

CAPACS Chennai Corporation AIDS Control Society

CCOORR Christian Council for Rural Development and Research

CI Confidence Interval

CSW Commercial sex workers

DFID Department for International Development (India)

EOS Economies of scale index

INR Indian Rupees

SACS State AIDS Control Society

STI Sexually transmitted infections

TNSACS Tamil Nadu State AIDS Control Society

VCT Voluntary counselling and testing

## Competing interests

The author(s) declare that they have no competing interests.

## Authors' contributions

LG led the design of the study, carried out the data collection and analysis and wrote the paper. LK supported the design of the study and supervised the data collection. KH supervised the data analysis. LK and KH provided critical input to the drafting of the paper and have both given approval of the final version.
